# Plastic or Fibrinous Bronchitis

**Published:** 1894-01-13

**Authors:** Alexander Cook

**Affiliations:** Cardiff


### PLASTIC OR FIBRINOUS BRONCHITIS.

With Notes of a Case. By Alexander Cook, M.D.Glasg., L.R.C.P.Edin., &c., Cardiff.

"This is one of the rarest diseases that are known to physicians" (C. Hilton Fagge), "an affection of great rarity" (Walshe). The experience of Sir Thomas Watson was remarkable, in having had under his own observation five well marked examples. This singular affection is certainly nothing else than bronchitis anatomically; but it has characters so peculiar that from a clinical point of view it would be absurd to group it with the ordinary catarrhal forms. It consists in the exudation of a fibrinous material from the walls of the air passages, which forms casts of their channels. A similar exudation may occur by extension downwards from the larynx in diphtheria, and by extension upwards from the alveoli in pneumonia, but these are entirely different, both in their symptoms and pathology; casts may also form as the result of hæmoptysis, but these also are very different. Cases were recorded by the younger Bartholin Cheselden, De Haen, Morgagni, Hunter, Cheyne of Dublin, and Stokes. Most of the early cases—1690-1730—were published in the "Philosophical Transactions," and Dr. Peacock gives a summary of thirty-four cases by various authors in the fifth volume of the "Transactions of the Pathological Society of London."

*Anatomical Characters.*—Plastic bronchitis is characterised by the formation of a peculiar exudation, of which the patient expectorates masses rolled up into a sort of ball, with a good deal of mucus and blood covering it, which is easily removed by floating it in water, when it can be seen that it is a complete cast of the bronchial tree. The colour of the cast is whitish, yellow, or grey. It is tough and elastic, and made up of a number of concentric laminæ, separated here and there by narrow spaces, and with a more or less definite central canal; the laminated structure affords a distinction from the branching clots, which are sometimes formed in the air passages as the result of hæmorrhage, and which are quite homogeneous.

The casts in fibrinous bronchitis, when examined microscopically, are seen to consist of a hyaline base in which are embedded large numbers of leucocytes; they seldom contain red blood corpuscles in any quantity. The length of a cast is usually from one and a half to two inches, but may be occasionally longer; the diameter of the thickest part is generally from an one-eighth to one-sixth of an inch, they are generally from tubes of the third or fourth magnitude. In fatal cases it is not usually found that the tubes which have poured out the fibrinous exudation show any marked morbid changes. The mucous membrane is sometimes reddened, sometimes pale and healthy looking, the pulmonary alveoli are usually unaffected, but they may be collapsed.

*Symptoms.*—The expectoration of casts of the bronchial tubes is generally attended with some cough and dyspnœa, the occurrence of which may be the first indication that the patient is otherwise than well; sometimes the disease sets in with rigors, loss of appetite, thirst, oppression of the chest and feverishness, then a hard dry cough appears, which may cause great suffering.

The breathing becomes rapid, forty or more in the minute, and may be attended with the greatest anguish as of impending suffocation, there is great lividity and a small hard pulse, there may be a feeling of soreness within the chest, but the attack is more distressing than acutely painful, at first nothing is expectorated or only a little mucus, usually a cast is detached and got rid of within a few hours, which generally at once relieves the cough and dyspnœa. Hæmoptysis occasionally occurs in small quantity during the paroxysm, but is at least quite as often absent as present.

*Physical Signs.*—Examination of the chest throws little light upon cases of plastic bronchitis. In practice, it scarcely ever happens that any suspicion of the real nature of the case arises, until a cast has actually been expectorated. There is not usually any change in the percussion sound, but Dr. Walshe says that he has had repeated occasion to observe dulness as complete as that of pneumonic consolidation dependent upon collapse of the lung substance. When there is extensive blocking of the tubes, the movements of the corresponding side of the chest may be distinctly impeded, rales are sometimes audible over the affected part of the lung, especially when the cast is becoming loose. The expectoration of a single cast very rarely brings an attack to an end, after some hours the cough and dyspnœa return and are followed by the appearance of another cast, this process is usually repeated once in 24 or 48 hours for several days, and then the process slowly subsides, small pieces being spat up at intervals.

*Prognosis.*—The expulsion of these large masses is attended with a considerable element of danger, more than one case has been recorded of death from dyspnœa, but as a rule the prognosis is favourable, a fatal result more often depending on some other organic disease. When an attack has passed off leaving the patient apparently well, it does not follow that the disease is at an end, for it is liable to return again and again sometimes during a long period.

*Ætiology.*—With regard to the causes of plastic bronchitis scarcely anything can be said. It is much more common in males than in females. Of 55 cases collected by Peacock and Fagge 42 were in males and 13 in females; of 58 cases collected by Biermer 39 were in males and 19 in females—or a percentage of males of 77 and 67 respectively. A remarkable circumstance, says Fagge, and all the more striking because of the extreme rarity of the disease is its occurrence in different members of the same family. Fuller met with it in two sisters and Sir Thomas Watson in two brothers, both of whom were affected within a twelvemonth. Riegel says that, like acute pneumonia, it is most apt to occur towards the end of spring, when there are great daily variations of temperature. There seems to be no reason to suppose that this remarkable affection is at all related to phthisis.

*Treatment*—Seems generally to be altogether ineffectual as far as a permanent cure is concerned, but is of considerable value during the paroxysm. Waldenberg saw a case in a girl aged eight and a-half years, who for more than four years had been coughing up fibrinous masses at intervals of a few days, and in whom a whey cure and the daily inhalation of lime water succeeded in arresting the disease in six or seven weeks. A spray of lime water or a solution of an alkaline carbonate should always be employed in plastic bronchitis; the only doubt is whether they reach the lower air passages in sufficient quantity. Emetics appear to be useful, probably it is best to use apomorphia hypodermically. Biermer recommends an active mercurial treatment, others have prescribed iodide of potassium with apparent advantage. Dr. Walshe believes that neither inhalation of iodine nor exhibition of alkaline remedies, nor the best general health, nor the most favoured climates have the least effect in preventing or curing the attacks.

The following are the notes of a case which occurred in my practice, with observations thereon:—

Mrs. D——, aged 52 years, housewife, sent an urgent message for me at half-past eleven p.m. on the night of October 1st, 1888. I found her standing at the side of her bed, supporting herself by the end of it, straining and gasping for breath, and scarcely able to speak or answer questions. The lips were livid, the skin hot and dry. She jerked out in a spasmodic way between her gasps for breath; that she was choking and was going to die, and complained of great thirst and a feeling of suffocation, and she looked very seriously ill.

*Previous History.*—She had lived for years in the same house, which is comfortable, clean, and healthy, and had always had plenty of good food and clothing; general habits were active and regular; she took very little stimulant.

*Previous Health.*—She had had small-pox when she was three or four years old. Eighteen years ago, when she was about 34 years of age, and immediately after her last confinement, she had something in her throat which she could not get up, but after rubbing well outside with castor oil, which her nurse recommended her to do, it came up. She did not notice what she expectorated, but it felt like thick phlegm. She had had no recurrence of the trouble till the present attack. She never had rheumatism nor hæmoptysis.

*History of Present Attack.*—The patient felt perfectly well and as usual on the morning of the day on which she was attacked. She had to go to town, a distance of a mile or so, in the afternoon, and as it was a cold day, with east wind, she wrapped herself well up. While she was out she felt chilly, and had a slight shivering and nasty headache. She got home about tea time, five o'clock, and the shivering got worse. A hard, dry, troublesome cough came on, with gradually increasing difficulty in breathing, till eleven p.m., when she sent for me, she was feeling very ill indeed, and, as she expressed it, felt like dying.

*Family History.*—Father lived to 52, died of aneurism, the result of an accident. Mother lived to 47, died of apoplexy. Brothers (two)—one died in infancy, the other, aged 51, is alive and well. Sisters (one) died in infancy. Both grand parents lived to a good old age, no history of consumption, rheumatism, or heart disease, nor anything like the present attack in either her father or mother's family.

I found her standing and holding on to the bed gasping for breath. Lips were livid, skin dry and hot. Temperature, 103 deg. F. She looked anxious, and had frequent hard, dry cough, but no expectoration, was well nourished, large framed, but not very stout. She looked her age, was perfectly intelligent, and complained of headache, rigors, and choking sensation, and a feeling of wanting to get something up, and said she would like to vomit. She answers questions in the middle of a gasp for breath. The pulse is small, hard, and regular, about 100 to the minute. The heart sounds were normal, also the situation of the apex beat. The respirations laboured, and about 40 to the minute. There was no pain in the chest, which was well formed, but there was much less movement on the left side than on the right. There was no dulness on percussion, but a few moist rales were to be made out on the left side. The lips were livid, the tongue dry; great thirst, but no appetite; urine high coloured, spec. gr. 1,025; no albumen, no sugar. A slightly disagreeable odour from the breath.

*Treatment.*—A fire was put in the room, and patient got into bed, but we had to prop her up with pillows, get the bronchitis kettle on the fire, and steam the room well with terebine. I also gave her terebine as an inhalation, next applied a hot linseed meal poultice with turpentine to the chest over the part where she complained of feeling the obstruction, then gave her two grains of tartar emetic, followed by a large amount of warm water, but it had no effect; repeated the dose and more water in half an hour, but still no effect; she continued to inhale the terebine, and felt slightly easier in her breathing, but still felt the feeling of suffocation and as if she wanted to get something up; so in about an hour I gave her apomorphia hypodermically in doses of 1-10th of a grain, with an interval of ten minutes between the dose, and soon after the third dose she vomited copiously, the vomit consisting largely of the warm water she had taken with a considerable amount of thin mucus, and a small, more firm, and consistent part, which proved on examination to be a very distinct cast of a bronchial tube, greyish yellow in colour, about 1½ inches in length and 1-6 in. broad, showing a distinct bifurcation; it had a slightly laminated structure, with a distinct central cavity filled with air bubbles and mucus. Under the microscope it was found to consist of a hyaline base, with leucocytes embedded in it, but no blood-globules, fat, or crystals.

Almost immediately she had got this up she expressed herself as feeling better, and having lost the choking sensation, and in fact she rapidly began to look better, but said she felt tired and exhausted; after giving her some beef tea, which they had made, I left her about half-past one a.m., feeling fairly comfortable, and as if she would sleep. I saw her early again, about eight a.m., and found she had passed a good night. She said she had slept a few hours and felt much better; temperature was normal, pulse 70, much better, respiration easy, about 20 to the minute, bowels had acted freely. I put her on a light diet, and prescribed iodide of potash, which she continued to take for some considerable time, about a month. She continued to expectorate smaller pieces of cast and some mucus for the next few days, after which she said she felt quite well, and I saw her only occasionally for about a month, during which she took the iodide, and for the last fortnight of which she was out of doors, and seemed quite well.

Five years after almost to the day, I again saw her, when she informed me that about a week previously she had had a very slight recurrence of the cough, and thick expectoration, which came up comparatively easily. She described it as roundish balls of thick phlegm, but with no long pieces (casts). She felt slightly out of sorts at the time, but not ill. She is now quite well again.

*Observations.*—The appearance of the patient, the comparatively sudden onset, and the noisy and struggling breathing made one think of spasmodic asthma. The history of rigors, and the temperature, of pneumonia. But on a physical examination there were absolutely little or no signs, no pain, no dulness, and very few rales. Again, the great feeling of suffocation and as if something were in the throat and difficult respiration were not like pneumonia. And the fact that she had not swallowed anything which could stick in the larynx, with the previous history of obstruction to breathing sixteen years ago, all point to only one thing which it could well be—viz., plastic bronchitis; but this is a very rare disease, and, as Walshe points out, the diagnosis is most usually made only when the patient expectorates the casts; and so it was in this case. There is no sign of the disease in her relations or children, and she is over the age at which most of the patients have been whose cases have been recorded. The treatment adopted in this case at least cut short the attack, and gave speedy relief, by getting rid of the obstruction; and the after-treatment by iodide of potassium, I think, should largely be given the credit of keeping off another attack for so long a period as five years, and the fact that the recurrence even then was in such a mild form. The active treatment of the paroxyism by apomorphia, inhalation of terebine and steam, and the hot poultices, I think, undoubtedly loosened and got rid of the cast, and gave her immediate relief—a relief which, in the patient's own words, she could only compare to that experienced on the completion of a very bad confinement. When it was over she felt well.

